# Antibodies produced in vitro in the detection of periodontal bacteria by using surface plasmon resonance analysis

**DOI:** 10.1002/cre2.6

**Published:** 2015-10-27

**Authors:** Sravya Sowdamini Nakka, Johanna Lönn, Carin Starkhammar Johansson, Torbjörn Bengtsson, Fariba Nayeri

**Affiliations:** ^1^ The Institution for Protein Environmental Affinity Surveys PEAS Institut AB Linköping Sweden; ^2^ Clinical Research Center, School of Health and Medical Sciences Örebro University Örebro Sweden; ^3^ Centre for Oral Rehabilitation, Public Dental Health Care County Council of Östergötland Linköping Sweden; ^4^ Division of Infectious Diseases University Hospital Linköping Sweden

**Keywords:** Antibodies, B‐cell, plasma cell, periodontitis, *P*. *gingivalis*, surface plasmon resonance

## Abstract

*Porphyromonas gingivalis* (*P*. *gingivalis*) is a major etiological agent associated with periodontitis. This study aims to develop antibodies to *P*. *gingivalis* in vitro for real‐time detection of bacteria in clinical samples. Lymphocytes were isolated from whole blood of patient treated for periodontitis and were stimulated with *P*. *gingivalis* ATCC 33277. B‐cell maturation to long‐living antibody secreting‐plasma cells was studied using flow cytometry and immunofluorescence staining. The antibodies developed in vitro were immobilized onto a CM‐5 sensor chip of a biosensor to detect the presence of *P*. *gingivalis* in the gingival crevicular fluid of patients with periodontitis compared to periodontally healthy controls (*n* = 30). Surface plasmon resonance (SPR) analysis was performed to evaluate specific interactions of bacteria in samples with the immobilized antibodies. The results of SPR analysis were compared to the detection of *P*. *gingivalis* in the samples using DNA–DNA checkerboard hybridization technique. A clear and distinct change in lymphocyte morphology upon stimulation with *P*. *gingivalis* was observed. Anti‐*P*. *gingivalis* antibodies secreted by CD38+ plasma cells showed the presence of all the four IgG subclasses. The results of DNA–DNA checkerboard analysis were in agreement with that of SPR analysis for the detection of *P*. *gingivalis* in patient samples. Furthermore, incubation with anti‐*P*. *gingivalis* attenuated the bacterial response in SPR. The in vitro method for antibody production developed during this study could be used for an efficient real‐time detection of periodontitis, and the attenuating effects of in vitro antibodies suggest their role in passive immunization to prevent periodontitis and their associated risk factors.

## Introduction

*Porphyromonas gingivalis* is a gram‐negative, black‐pigmented anaerobic bacterium and a major etiological agent of periodontitis. Periodontitis is a chronic inflammatory polymicrobial condition leading to tissue destruction and tooth loss (Kadowaki et al*.,* 2007). Increased inflammatory mediators and evasion strategies of oral pathogens during periodontitis have been associated with increased risk for systemic diseases such as atherosclerosis, rheumatoid arthritis, diabetes, and kidney dysfunction (Hayashi et al*.,* 2010). *P*. *gingivalis* and other periodontopathic bacteria evade the host immune responses by a variety of mechanisms such as interfering with host mechanism to detoxify toxins, inhibit adherence and colonization, increase protease activity degrading host enzymes/proteins, block anti‐inflammatory cytokines, and prevent leukocyte migration to the infected site (Gupta and Gupta, 2014; Jain and Darveau, 2010). Virulence factors of *P*. *gingivalis* include their fimbriae, capsular polysaccharides, hemagglutinin, lipopolysaccharide, gingipains, outer membrane protein, antigen, and heat shock proteins (Holt et al*.,* 1999). Antigenic specificities are important to elicit antibody responses.

Previous studies have shown increased serum IgG antibodies in periodontal patients (Pussinen et al*.,* 2003). The importance of antibodies is to bind to the pathogen at antigen binding site to detoxify their toxins or opsonize or present to phagocytes (Gupta and Gupta, 2014). Several studies have been performed to investigate the role of serum IgG antibodies to the capsular antigen and fimbriae of *P*. *gingivalis* where antibodies were shown to enable phagocytosis to eliminate bacteria and thereby offer protection (Gupta and Gupta, 2014). However, *P*. *gingivalis* can degrade serum IgA antibodies as an evasion strategy to survive within the host (Sato et al*.,* 1987). Several studies have also suggested that elevated serum antibody levels in patients with periodontitis, correlating to alveolar bone loss, are a key factor associated with coronary heart diseases (Pussinen et al*.,* 2003).

IgG titers have been used as a diagnostic tool as an indicator of immune status of an individual and are used to study several diseases like rubella, measles, hepatitis B, and chicken pox (Brettschneider et al*.,* 2009; Rousseau and Hedman, 1988; Suga et al*.,* 1991). However, the sensitivity of conventional culture and polymerase chain reaction (PCR)‐based detection methods for bacterial screening in clinical investigations is still under debate. Diagnosis of periodontitis generally includes examining the health of gingival tissues (color, texture, bleeding on probing, and plaque), tooth mobility, tooth loss, and prevalence of periodontal pockets (Hefti, 1997). Elevated serum IgG levels during periodontitis were observed to decrease with decrease in bacterial count in periodontal pockets after treatment (Highfield, 2009) (Kudo et al*.,* 2012).

Efforts to understand the pathophysiology of various immune‐related diseases for developing efficient diagnostic methods have resulted in the production of monoclonal, polyclonal, and recombinant antibodies of different grades of specificity by stimulating the immune system in diverse animal models (Bradbury and Marks, 2004; Diano et al*.,* 1987; Jackson et al*.,* 1999). Furthermore, a specific antigen‐antibody interaction is used to develop ELISA kits (Invitrogen Life Technologies, Stockholm, Sweden) for clinical investigations (Joynson et al*.,* 1990).

Recent advances in science have led to improved technology that is mostly nucleic acid or antibody‐based methods for detection of bacteria. Currently improved PCR technology such as multiplex PCR, which could also screen mutations in detected bacterial genes (Hayden et al*.,* 2008) and qPCR (Bohaychuk et al*.,* 2007), has been used for rapid screening of bacteria with improved sensitivity compared to conventional methods. Broad spectrum and rapid detection, genotyping, and characterization could be performed using microarrays (McLoughlin, 2011) and next generation sequencing (Cummings et al*.,* 2010). Conventional culture‐based methods are time‐consuming, laborious, and less sensitive to detect microbes at species level (Park et al*.,* 2014). Although these advanced techniques have features to detect, characterize, and perform comparative genomics in a precise manner, the need of skilled‐technicians and high costs makes it less feasible to be used in hospitals (Park et al*.,* 2014).

Surface plasmon resonance (SPR) biosensors are used to study the interaction of analytes to specific ligands immobilized on gold‐coated dextran surface of a sensor chip using a label‐free technology in real time. An interaction of biomolecules causes change in angular position of the produced optical signal leading to a change in refractive index that could be measured in response units (RUs) using bia‐evaluation software (GE healthcare Ltd., Stockholm, Sweden) (Waswa, 2006). SPR analysis has been used widely to detect adulterants, additives, toxins, and pathogens in food samples (Bokken et al*.,* 2003; Nedelkov et al*.,* 2000; Tudos et al*.,* 2003), to study drug target interactions (Thurmond et al*.,* 2001) and to characterize the therapeutic role of antibodies and drugs (Gonzales et al*.,* 2002).

In this study, host lymphocytes were stimulated with *P*. *gingivalis* to develop antibodies in vitro, and anti‐*P*. *gingivalis* antibodies were used for immunodetection in clinical samples. Anti‐*P*. *gingivalis* antibodies were immobilized onto the sensor chip of an optical biosensor and the change in resonance units because of binding affinity of specific bacteria in samples to the immobilized antibodies was evaluated using SPR analysis to detect bacteria real time in clinical samples.

## Materials and Methods

### Sample collection and isolation of lymphocytes

Blood was collected in ethylenediaminetetraacetic acid (EDTA) vials (Venoject K2E, Leuven, Belgium) from a donor who is cured of periodontitis after treatment (Centre for Oral Rehabilitation, Public Dental Health Care, County Council of Östergötland, Linkoping, Sweden). Whole blood was diluted in 0.9% NaCl solution in a 1:1 ratio and was carefully layered onto 6.0 mL of lymphoprep solution (Axis‐shield PoC, Oslo, Norway) in two ficoll tubes (Thermofischer Scientific Inc., Stockholm, Sweden) for gradient centrifugation at 1800 *g* at 20 °C for 20 min. A clear distinct layer of lymphocytes was carefully pipetted out and was washed with phosphate‐buffered saline (PBS; pH 7.2; Apoteket AB, Linkoping, Sweden). The cells cultured in l‐15 medium containing l‐glutamine (ATCC, Boras, Sweden), supplemented with 10% fetal bovine serum (FBS; Sigma‐Aldrich, Stockholm, Sweden) and incubated at 37 °C overnight.

The regional ethical committee in Linköping approved the study (2010/307‐31), and all the patients participated in our study were informed with consent.

### Bacterial culturing and stimulation of lymphocytes

*Porphyromonas gingivalis* wild‐type ATCC 33277 (American Type Culture Collection, Manassas, VA), wild‐type W 50, E8 (W50 derived RgpA‐deficient and RgpB‐deficient) and K1A (W50 derived Kgp‐deficient) (kindly provided by Prof. M. A. Curtis, Molecular Pathogenesis Group, Queen Mary, University of London, UK), wild‐type 381, DPG‐3 (381 derived Fim A‐deficient), and KRX‐178 (381‐derived Mfa‐1‐deficient) (kindly provided by Prof. R. J. Genco, Department of Microbiology and Immunology, Buffalo University, NY) were grown in fastidious anaerobe broth (29.7 g/L, pH 7.2) under anaerobic conditions (80% N_2_, 10% CO_2_, and 10% H_2_) at 37 °C in a chamber (Concept 400 Anaerobic Workstation; Ruskinn Technology Ltd., Leeds, UK). The bacteria were washed and resuspended in Krebs–Ringer glucose buffer (120 mM NaCl, 4.9 mM KCl, 1.2 mM MgSO_4_, 1.7 mM KH_2_PO_4_, 8.3 mM Na_2_HPO_4_, and 10 mM glucose, pH 7.3).

Heat‐killed *P*. *gingivalis* was prepared by incubation at 80 °C for 20 min. A total of 10 μL of the heat‐killed suspension was spread on a fastidious anaerobe agar plate to ensure that the bacteria were killed and incubated at 37 °C for 5 days. Heat‐killed *P*. *gingivalis* ATCC 33277, 10^9^ cfu/mL was used to stimulate the lymphocytes and incubated at 37 °C. Fresh medium was supplemented at regular interval until 21 days.

### Flow cytometry analysis of lymphocytes

Flow cytometric analysis was performed to study the cell composition. The cells were centrifuged at 300 *g* (Eppendorf Centrifuge 5702R5, Germany) for 5 min, and the pellet was dissolved in 1000 μL of PBS upon discarding the supernatant. Cell composition was determined by mixing 100 μL of the sample with 20 μL antibody cocktail of CD19, CD3, CD16/56, and CD45 surface markers tagged with the fluorochromes allophycocyanin (APC), fluorescein isothiocyanate (FITC), phycoerythrin (PE), and Peridinin chlorophyll protein complex (PerCP) (BD Biosciences, Stockholm, Sweden) and incubated for 15 min in the dark. Red blood cells were lysed by adding 450 μL of lysing solution (2% Fetal calf serum (FCS; Sigma‐Aldrich, Stockholm, Sweden), 0.1% sodium azide solution in PBS pH 7.4). After 15 min of incubation, the cells were analyzed in BD FACS Canto (BD Biosciences, Stockholm, Sweden). The results were plotted on a logarithmic scale by gating CD45+ lymphocyte cells for analysis. Cells were analyzed 1 week after the stimulation with bacteria, whereas unstimulated cells were analyzed after 3 days. The unstimulated lymphocytes after 3 days did not contain sufficient cell counts for further analysis due to the absence of an activating agent. Therefore, lymphocytes were isolated and cultured from the blood of the same patient, as described previously, three days prior to flow cytometry analysis.

### Immunofluorescent staining and confocal microscopy

Cell suspensions stimulated by 2 and 3 weeks of incubation were centrifuged at 170 *g*, and the pellet was dissolved in 2 mL of fresh l‐15 medium containing 10% FBS. A total of 200 μL of 5 × 10^5^ cells/mL suspension was then cytocentrifuged at 600 rpm for 6 min. Paraformaldehyde (4%) was used to fix the cells for 2 h at room temperature. The fixed cells were washed four times with PBS at intervals of 5 min of incubation, blocked using 5% FBS for 1 h at room temperature, and then washed again using the same protocol. The cells were incubated with anti‐human CD38 antibodies (BD Biosciences, Stockholm, Sweden; diluted 1:100) overnight at 4 °C. The cells were washed with PBS as before and then incubated with fluorescein isothiocyanate conjugated‐goat anti‐mouse antibodies for 2 h at room temperature. The cells were washed four times with PBS and then counterstained with 1:200 dilution of 4ʹ,6‐diamidino‐2‐phenylindole (DAPI) (Sigma‐Aldrich, Stockholm, Sweden) for 3 min. After washing four times, the cells were analyzed in a laser‐capture microdissection (LCM) Zeiss confocal microscope (Department of Pathology, Linköping University, Sweden).

### Antibody recovery

After 3 weeks, culture medium was transferred into sterile 15 mL centrifuge tubes (Greiner Bio‐One, Germany) and centrifuged at 3500 *g* for 10 min to pellet the cells. The supernatant was filtered using 0.45 µm sterile filters (Thermofisher Scientific, Stockholm, Sweden) and centrifuged using 100 KDa Amicon microcon cutoff filters (Millipore, Molsheim, France) at 3500 *g* for 60 min. The supernatant containing antibodies to *P*. *gingivalis* with molecular weight >100 KDa was collected, and the sterile was filtered with 0.2‐µm sterile filters (Thermofisher Scientific, Stockholm, Sweden) to be stored at −50 °C.

### Sodiumdodecyl sulfate polyacrylamide gel electrophoresis

Standard sodiumdodecyl sulfate polyacrylamide gel electrophoresis was performed to visualize the antigen‐antibody interaction as an alternative method for showing the specificity of antibodies to the antigens. Heat‐killed *P*. *gingivalis*, which was used to stimulate the host lymphocytes, was incubated with both anti‐*P*. *gingivalis* antibodies produced in vitro and anti‐*Escherichia coli* antibodies (Swed Diagnostics, Stockholm, Sweden).

All the prepared samples were diluted at 1:4 ratio with PBS (pH 7.4, Sigma‐Aldrich, Stockholm, Sweden) and were centrifuged at 3450 *g* for 10 min after 24 h of incubation. Supernatants were centrifuged in 100 KDa centrifuge filters (Millipore, Stockholm, Sweden) and were heated with equal volume of Laemmli buffer for 5 min. Samples were run in sodiumdodecyl sulfate polyacrylamide gel electrophoresis TGX precast gels (Bio‐rad, Stockholm, Sweden).

### IgG subclass assay

IgG subclass analysis was performed on the antibodies produced in vitro using an ELISA kit. Pre‐coated ELISA plates and the reagents were prepared according to the manufacturer's instructions. In vitro antibodies produced against *P*. *gingivalis* were diluted with ELISA diluent in a 1:4 ratio. Peroxidase anti‐human IgG solution was used as secondary antibody, and 3,3ʹ,5,5ʹ‐tetramethylbenzidine substrate solution was used for the chromogen reaction. The results were read at 450 nm within an hour after adding the stop solution in an ELISA microreader (Expert 96 ELISA reader, GmBH, Austria). Human serum provided by the manufacturer was used as control, and concentrations were calculated from the standards.

### Surface plasmon resonance

Anti‐*P*. *gingivalis* antibodies were immobilized on a CM 5 sensor chip of a Biacore® 1000 (GE Healthcare Ltd, Sweden) to investigate specific antigen‐antibody interactions using the amine coupling method. Immobilization was carried out in three steps. First, the carboxymethylated dextran‐coated channel surface on chip was activated by injecting an *N*‐hydroxysuccinimide and *N*‐ethyl‐Nʹdimethyl aminopropyl carbonamide hydrochloride (GE Healthcare Ltd, Sweden) mixture (1:1 ratio). Anti‐*P*. *gingivalis* antibodies were diluted with acetate (pH 4.5), which binds to the activated surface of the chip on injection. Finally, the sensor surface was deactivated by injecting 70 μL ethanolamine (GE Healthcare Ltd, Sweden), preventing the binding of other particles to the sensor surface.

The immobilized antibodies were tested by running positive and negative controls. Anti‐human IgG (Sigma‐Aldrich, St Louis, MO) and heat‐killed *P*. *gingivalis* used to stimulate the lymphocytes earlier were used as positive controls. Several ATCC bacterial strains (CCUG, Gothenberg, Sweden) and anti‐guinea pig IgG (Sigma‐Aldrich) were used as negative controls. The response obtained with SPR was measured in RUs using Bia‐evaluation software (GE Healthcare Ltd., Sweden).

### Detection of *Porphyromonas gingivalis* in vitro and in clinical samples

Plasma samples from healthy blood donors were collected and incubated with 10^8^ cfu/mL of wild‐type and fimbriae mutants of *P*. *gingivalis*. Samples were diluted 1:20 in PBS 7.4 (Sigma‐Aldrich, Stockholm, Sweden), and a set of these was incubated with anti‐*P*. *gingivalis* antibodies overnight. Samples, with and without antibody incubation, were run on the chip immobilized with anti‐*P*. *gingivalis* antibodies at a flow rate of 5 μL/min.

Gingival crevicular fluid (GCF) samples were collected from patients (*n* = 30) with severe, untreated periodontitis at the Department of Periodontology, Centre for Oral Rehabilitation in Linköping, Sweden, and from a group of age and sex‐matched periodontally healthy controls (*n* = 30).

Samples were obtained from the deepest periodontal pocket in each quadrant or from the mesial site of all first premolars in control subjects without deep pockets. Supragingival plaque was removed without salivary contamination, and the tooth was allowed to air dry. Periopaper strips were inserted to a depth of about 1–2 mm subgingivally to collect GCF, and the volume of GCF absorbed by the strips was determined using a Periotron 8000 (Oraflow Inc, NY), which was calibrated as described previously (Chapple et al*.,* 1999). GCF from four periopaper strips per patient was pooled into diluent buffer (Quantikine Human HGF immunoassay, R&D Systems, Minneapolis, MN) and was frozen at −20 °C until used.

Samples were thawed before use and were diluted in 1:20 ratio with sterile PBS (Sigma‐Aldrich, Stockholm, Sweden). Samples were run on the chip immobilized with anti‐*P*. *gingivalis* antibodies at a flow rate of 5 μL/min.

### DNA–DNA checkerboard hybridization analysis

Subgingival microbial samples were collected from the same four sites as GCF sampling. Supragingival plaque was removed, and the root surface was allowed to air dry. The bacterial sample was collected by inserting a Sterile‐endodontic paper point inserted into the periodontal pocket was used to collect subgingival bacterial samples from the same four sites where GCF was collected for 20 sec, which was then transferred to a sterile test tube. Samples were processed at the Department of Oral Microbiology and Immunology, University of Gothenburg, Sweden, and analyzed for the presence *P*. *gingivalis*, using the DNA–DNA checkerboard hybridization technique with a detection limit of at least 10,000 cells (Socransky et al*.,* 1994). The samples were also included in our previous studies (Lonn et al*.,* 2014).

### Real‐time polymerase chain reaction

Real‐time PCR analysis was performed to validate SPR analysis method of detection of *P*. *gingivalis* in GCF samples. Samples that showed different responses in DNA–DNA checkerboard analysis and SPR methods were chosen and were compared to the healthy controls. *P*. *gingivalis* 16S rRNA forward primer sequence of TGTAGATGACTGATGTGTAAAACC and reverse primer sequence of ACGTCATCCCCACCTTCCTC was used. SYBR Green (Maxima® SYBR Green/ROX qPCR Master Mix, Fermentas, Sweden) was used, and the PCR conditions were set to initial denaturation at 95 °C for 10 min followed by 95 °C, 15 sec for 40 cycles, and then 60 °C, 60 sec in 7900 HT real‐time PCR instrument (Applied Biosystems).

### Transmission electron microscopy

Transmission electron microscopy was performed to study the binding affinity of anti‐*P*. *gingivalis* to *P*. *gingivalis*. *P*. *gingivalis* diluted 1:20 in PBS was incubated with anti‐*P*. *gingivalis* antibodies diluted 1:100 in PBS. Excess antibodies were washed off with PBS, and gold‐labeled anti‐human IgG was added in the ratio 1:1000 (Nano Gold Probes, NY). The mixture was incubated at 37 °C for 1 h. Excess antibodies were washed with PBS and fixed with 1% osmium tetroxide (Sigma‐Aldrich, Stockholm, Sweden) until mounting on 200 µm mesh formvar‐coated copper grid (Agar Scientific, Stansted, UK). Uranyl acetate (2%, Sigma‐Aldrich, Stockholm, Sweden) was used for staining before examining in JEOL 1230 transmission electron microscope (Department of Pathology, Linkoping University, Sweden).

### Statistical analysis

Chi square analysis was performed to compare the results obtained from the two methods of SPR analysis and DNA–DNA checkerboard analysis, SPR analysis and reverse transcription‐PCR (RT‐PCR) by using Graph pad prism, version 5.0 (GraphPad Software, Inc., California, USA). From the chi square distribution table for degree of freedom 1 and *P* > 0.05 was considered as a rejection of null hypothesis (Fisher RAaY, 1963) with no significant differences between the results obtained by the two methods being analyzed.

## Results

### Lymphocyte activation by *Porphyromonas gingivalis*

**Figure 1:**
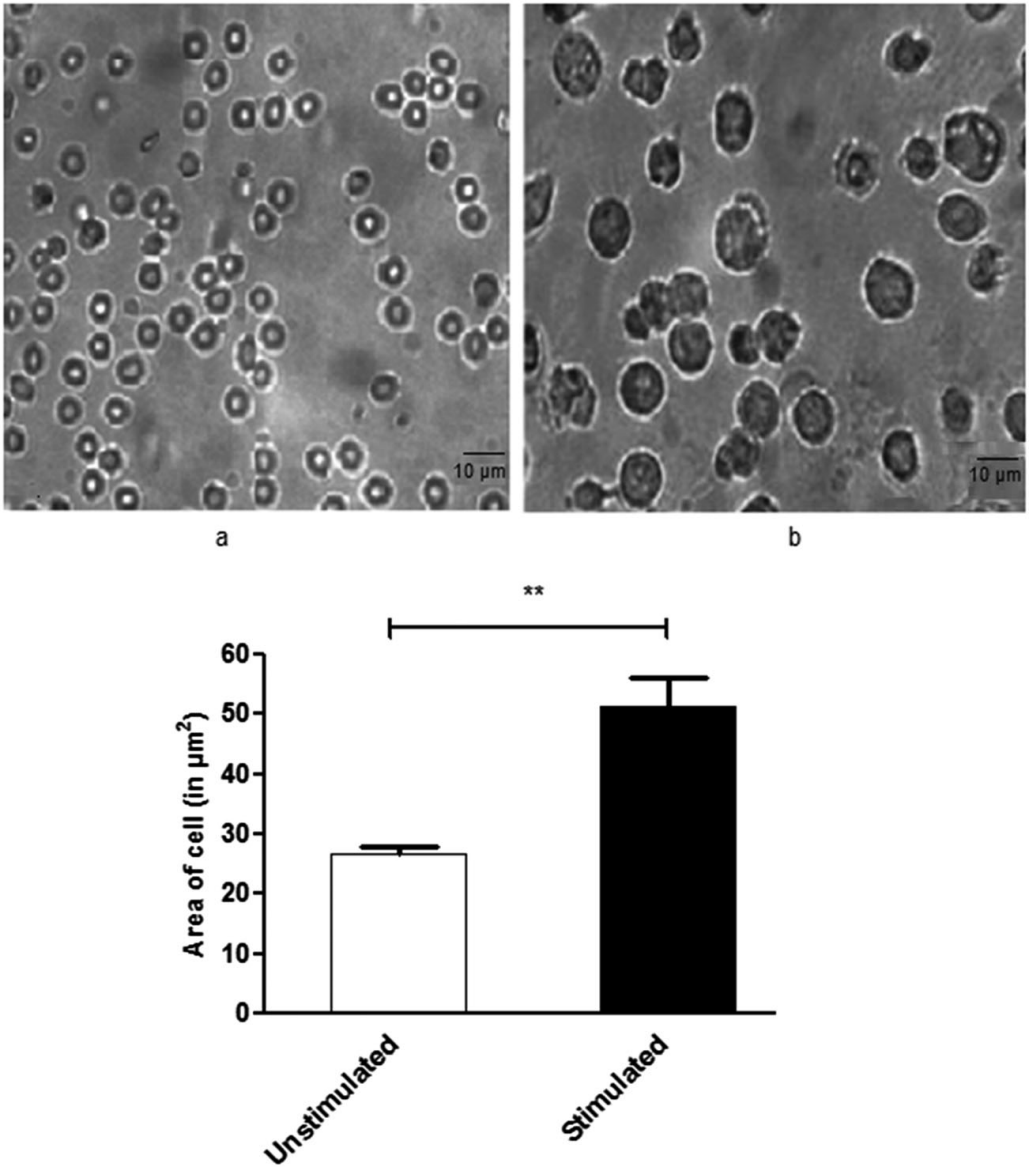
Microscopic analysis of lymphocytes. An immune response to heat‐killed *Porphyromonas gingivalis* in stimulated lymphocytes was observed by light microscopy at 40× resolution. Stimulated lymphocytes (b) were observed to increase in size and also showed changes in granularity than unstimulated lymphocytes (a) from the same donor who previously had encountered *P*. *gingivalis* infection. image j software was used to measure the significant changes in cell size upon stimulation (*P* < 0.005; two‐tailed *t*‐test).

Activation of lymphocytes on stimulation with heat‐killed *P*. *gingivalis* was studied using light microscopy. Lymphocytes stimulated with *P*. *gingivalis* exhibited morphological changes, such as increased size and granulation when compared to unstimulated cells (Fig. 1).

**Figure 2:**
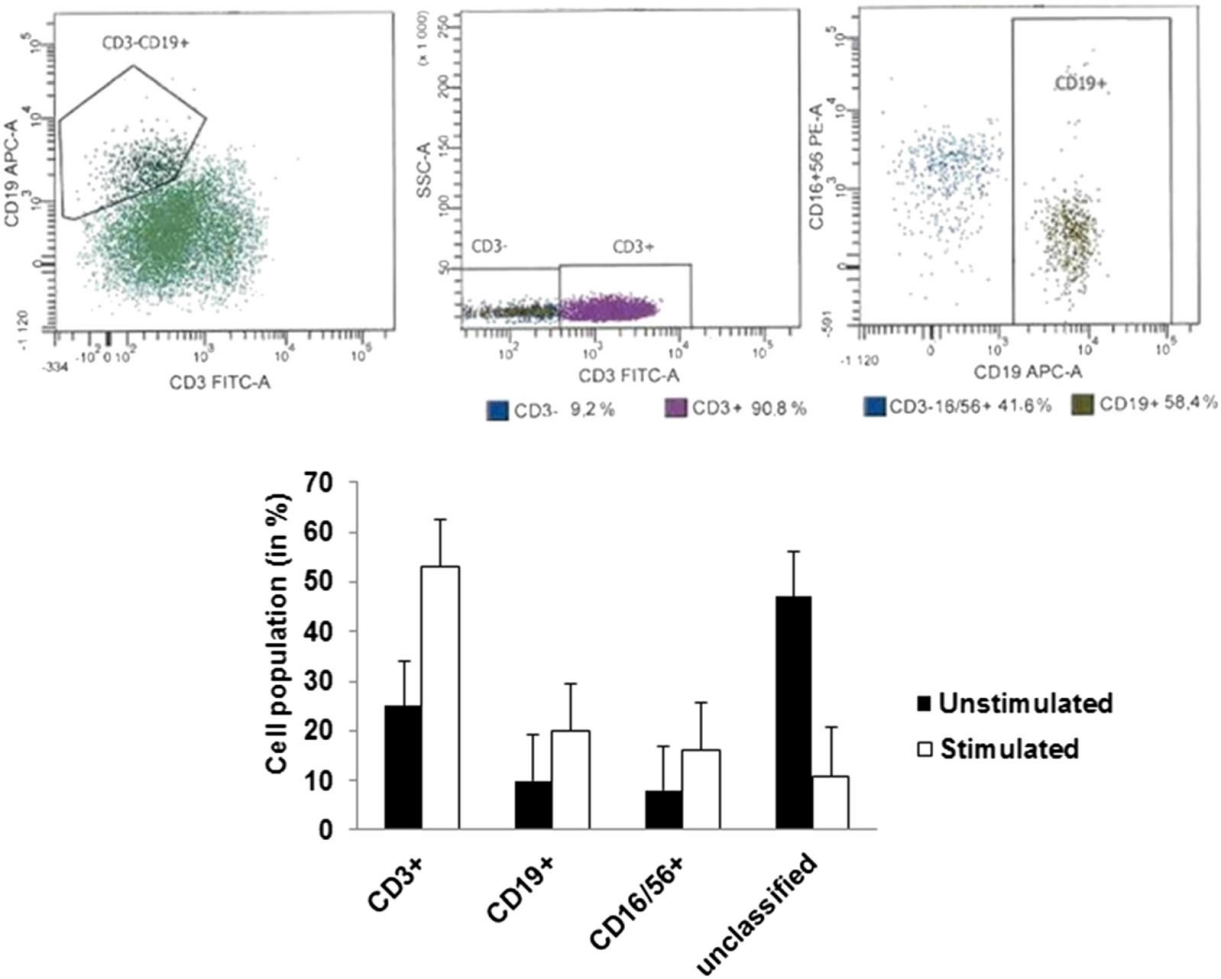
Flow cytometric analysis of lymphocytes. Lymphocyte population on stimulation with heat‐killed *Porphyromonas gingivalis* was compared to unstimulated lymphocytes using flow cytometry. CD45+ lymphocytes were gated, and lymphocyte population was analyzed. An increase in CD19+ B‐cells, CD3+ T‐cells, and CD16/56+ NK cells was observed 2 weeks after stimulation with heat‐killed *P*. *gingivalis*.

**Figure 3:**
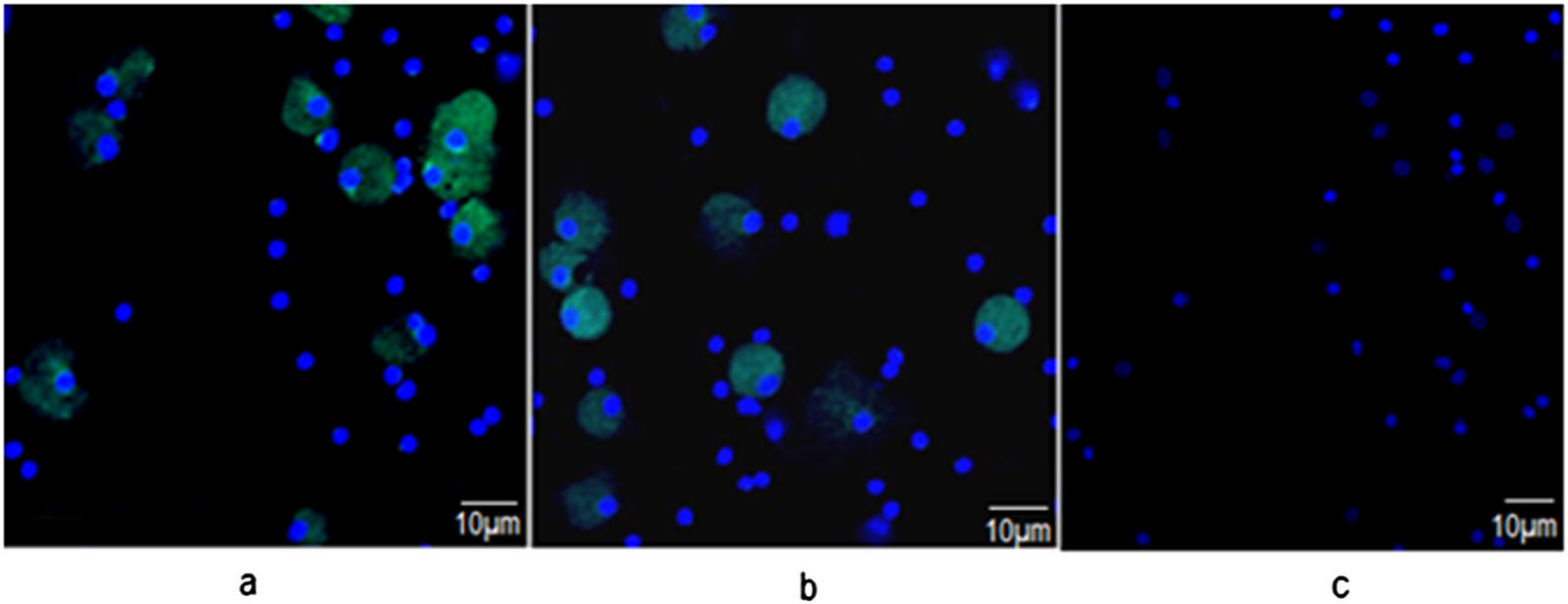
Immunofluorescent staining of long‐living plasma cells in vitro. Antibody‐secreting plasma cells (green) stained with anti‐CD38 antibodies 2 (a) and 3 weeks (b) after stimulation with *Porphyromonas gingivalis*. Fluorescein isothiocyanate (FITC) goat anti‐mouse antibodies were used as secondary antibodies to visualize the plasma cells by confocal microscopy at 20× resolution. Cells were counterstained with 4ʹ,6‐diamidino‐2‐phenylindole (blue), and FITC goat anti‐mouse antibodies without anti‐CD38 antibody were used as a negative control (c).

Composition of immune cells in the activated lymphocyte population was studied using flow cytometry analysis. All CD45+ cells were gated to identify the population of lymphocytes in the samples upon stimulation with *P*. *gingivalis* ATCC 33277. Significant increase in CD19+ (B‐cells), CD3+ (T‐cells), and CD16/56+ (NK‐cells) cells was observed in lymphocyte samples stimulated with *P*. *gingivalis*, compared to unstimulated lymphocytes (Fig. 2). B‐cell maturation into antibody secreting plasma cells expressing CD 38 was visualized using confocal microscopy on the 14th and 21st day after stimulation (Fig. 3).

### Antibody analysis

**Figure 4:**
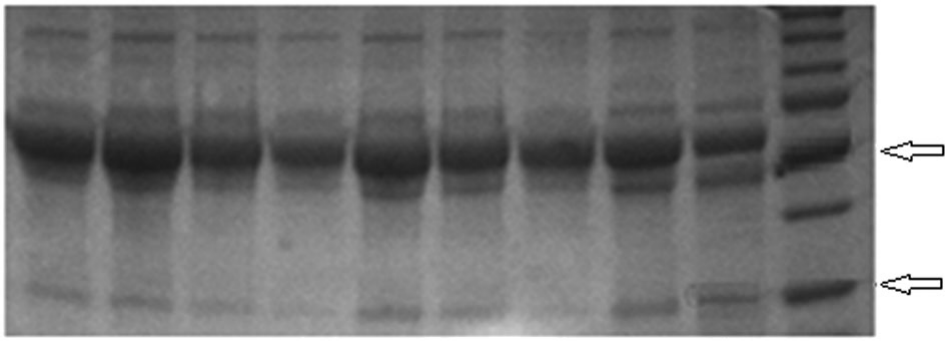
Sodiumdodecyl sulfate polyacrylamide gel electrophoresis (SDS‐PAGE) of the anti‐*Porphyromonas gingivalis* antibodies. SDS‐PAGE analysis of anti‐*P*. *gingivalis* antibodies showing IgG fragments. Antibodies were diluted in phosphate‐buffered saline in 1:5 ratio and were heated at 92 °C for 5 min with *β*‐mercaptoethanol and Laemmli buffer. Denatured samples (*n* = 9) were run in pre‐cast SDS gels at 65–115 V. Protein ladder was used for reference. Heavy chain bands were obtained at 50 KDa, while light chain bands were obtained at 25 KDa.

**Figure 5:**
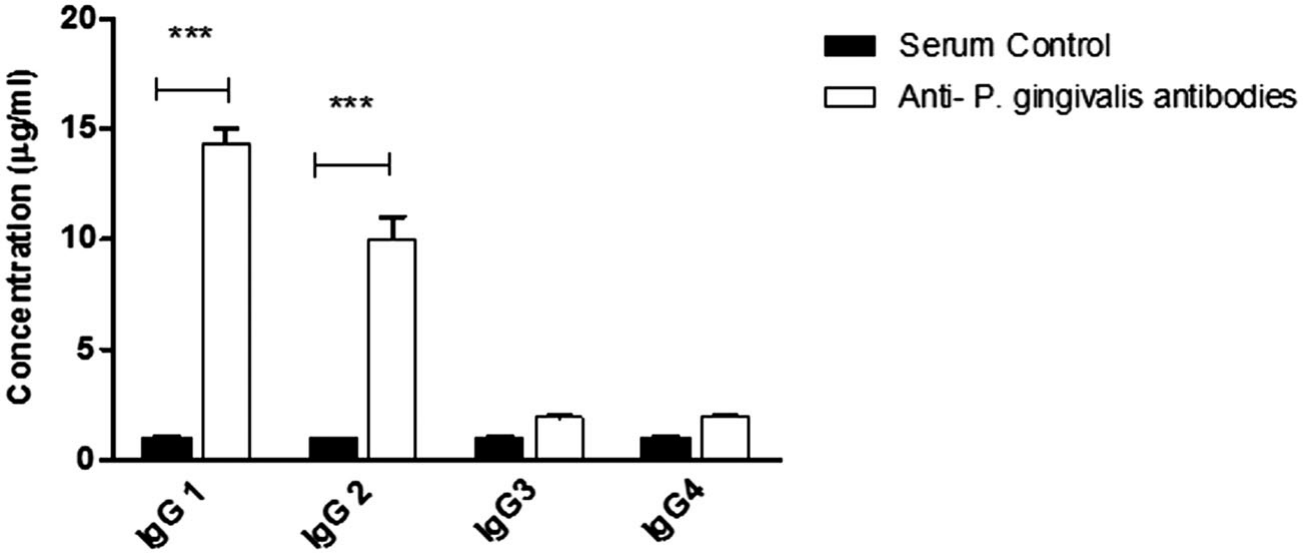
Presence of IgG subclasses in anti‐*P. gingivalis* antibodies. Concentrations of IgG subclasses in each of the antibodies produced in vitro were determined using IgG ELISA subclass assay. Human serum control provided by the manufacturer was used as control. The results presented are the mean values of samples in duplicates. *P*. *gingivalis*, *Porphyromonas gingivalis*.

Sodiumdodecyl sulfate polyacrylamide gel electrophoresis analysis of anti‐*P*. *gingivalis* antibodies yielded heavy and light chain bands of IgG at 50 and 25 KDa, respectively (Fig. 4). IgG subclass assay was performed to study the composition of IgG secreted in response to *P*. *gingivalis*, and concentrations of IgG1, IgG2, IgG3, and IgG4 were measured using human serum as a control (Fig. 5). IgG1 and IgG2 were the most dominant forms of anti‐*P*. *gingivalis* antibodies.

### Specificity test of antibodies with surface plasmon resonance and electron microscopy

The antibodies produced in vitro were subjected to specificity test using SPR analysis. Immobilization response was about 6400 RU and 6258 RU (1RU = 1 pg/mm^2^ concentration of protein) for antibodies produced in vitro and commercially purchased antibodies, respectively. Anti‐*P*. *gingivalis* antibodies produced in vitro were positive for anti‐human IgG and heat‐killed *P*. *gingivalis* ATCC 33277, respectively, whereas a negative response was obtained to all of the other eight bacterial strains tested as well as to anti‐guinea pig IgG (Table 1). These data show a very high specificity of anti‐*P*. *gingivalis* antibodies in SPR analysis. The commercial antibodies were not as highly specific as the antibodies produced in vitro although they showed similar responses to controls but were cross reactive with the other bacteria tested (Table 2).

**Table 1:** Specificity test of anti‐*P*. *gingivalis* antibodies in Biacore® 1000

Samples	Anti‐*P*. *gingivalis* channel
*Staphylococcus aureus*	0
*Staphylococcus epidermidis*	0
*Enterococcus faecalis*	0
*Pseudomonas aeruginosa*	0
*Bacteroides fragilis*	0
*Clostridium perfringens*	0
*Prevotella oralis*	0
*Escherichia coli*	0
*P*. *gingivalis*	101
Anti‐guinea pig IgG	0
Anti‐human IgG	882

*P*. *gingivalis*, *Porphyromonas gingivalis*; SPR, surface plasmon resonance.

Specificity of anti‐*P*. *gingivalis* antibodies immobilized on sensor chip was studied using SPR analysis. Heat‐killed *P*. *gingivalis* and anti‐human IgG were used as positive controls, while other bacteria and anti‐guinea pig IgG were used as negative controls. Response obtained with each of the samples in SPR analysis is presented in response units.

**Table 2:** SPR analysis to test the specificity of in vitro and in vivo antibodies

Samples	A	B
*Staphylococcus aureus*	−	−
*Staphylococcus epidermidis*	−	−
*Pseudomonas aeruginosa*	−	+
*Bacteroides fragilis*	−	−
*Clostridium perfringens*	−	−
*Prevotella oralis*	−	−
*P*. *gingivalis*	+	+
*Escherichia coli*	−	+
*Enterococcus faecalis*	−	+
Anti‐guinea pig IgG	−	−
Anti‐human IgG	+	+

SPR, surface plasmon resonance; *P*. *gingivalis*, *Porphyromonas gingivalis*.

+ Specific interaction with the antibodies immobilized on chip.

− No interaction with the immobilized antibodies.

CM‐5 sensor chip immobilized with antibodies produced in vitro (A) during the study was compared to the commercially available polyclonal antibodies in vivo produced in rabbit (B). Specific interaction of *P*. *gingivalis* to their antibodies developed in vitro and in vivo, immobilized on two different channels of the CM‐5 sensor chip, was studied by measuring the response obtained using bia‐evaluation software. Sensitivity and specificity of antibodies developed in vivo were found to be 100% and 60%, while antibodies produced in vitro showed 100% specificity and sensitivity in SPR analysis.

**Figure 6:**
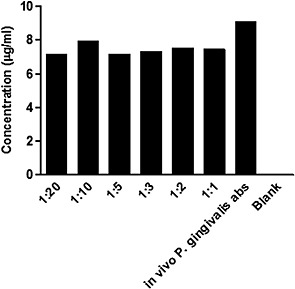
Anti‐*Porphyromonas gingivalis* antibodies binding to *P*. *gingivalis*. *P*. *gingivalis* ATCC 33277 (10^8^ cfu/mL) was coated on 96 well ELISA plate. Concentrations of anti‐*P*. *gingivalis* antibodies prepared in different dilutions were measured at Optical density (OD) 450 nm using Horseraddish peroxidase (HRP) conjugated‐anti‐human IgG as secondary antibodies. Rabbit anti‐*P*. *gingivalis* antibodies (Santa Cruz, USA) diluted as 1:5 in phosphate‐buffered saline (PBS) and human IgG (ELISA subclass assay with equal amounts of all 4 IgG subclasses) were use as controls. PBS diluent was used as blank.

Concentrations of different dilutions of anti‐*P*. *gingivalis* antibodies binding to *P*. *gingivalis* (10^8^ cfu/mL) coated plates was measured using ELISA. It was found that anti‐*P*. *gingivalis* antibodies were able to bind specifically to *P*. *gingivalis* at all the concentrations tested (Fig. 6).

**Figure 7:**
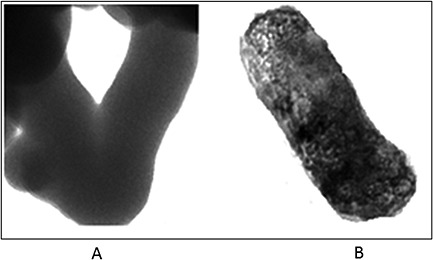
Antigen‐antibody interaction was visualized using transmission electron microscopy. Specific interaction of *Porphyromonas gingivalis* to their antibodies was studied in transmission electron microscopy. *P*. *gingivalis* without antibodies (a) and anti‐*P*. *gingivalis* antibodies developed during the study bound specifically to *P*. *gingivalis* bacteria upon 24 h of incubation (b) were seen at 100,000×.

Heat‐killed *P*. *gingivalis* ATCC 33277 was visualized in transmission electron microscope. *P*. *gingivalis* incubated with anti‐*P*. *gingivalis* antibodies revealed a heterogenous surface indicating a specific binding of the antibodies when compared to smooth surface of *P*. *gingivalis* without antibodies (Fig. 7).

### Detection of *Porphyromonas gingivalis* in clinical samples

**Figure 8:**
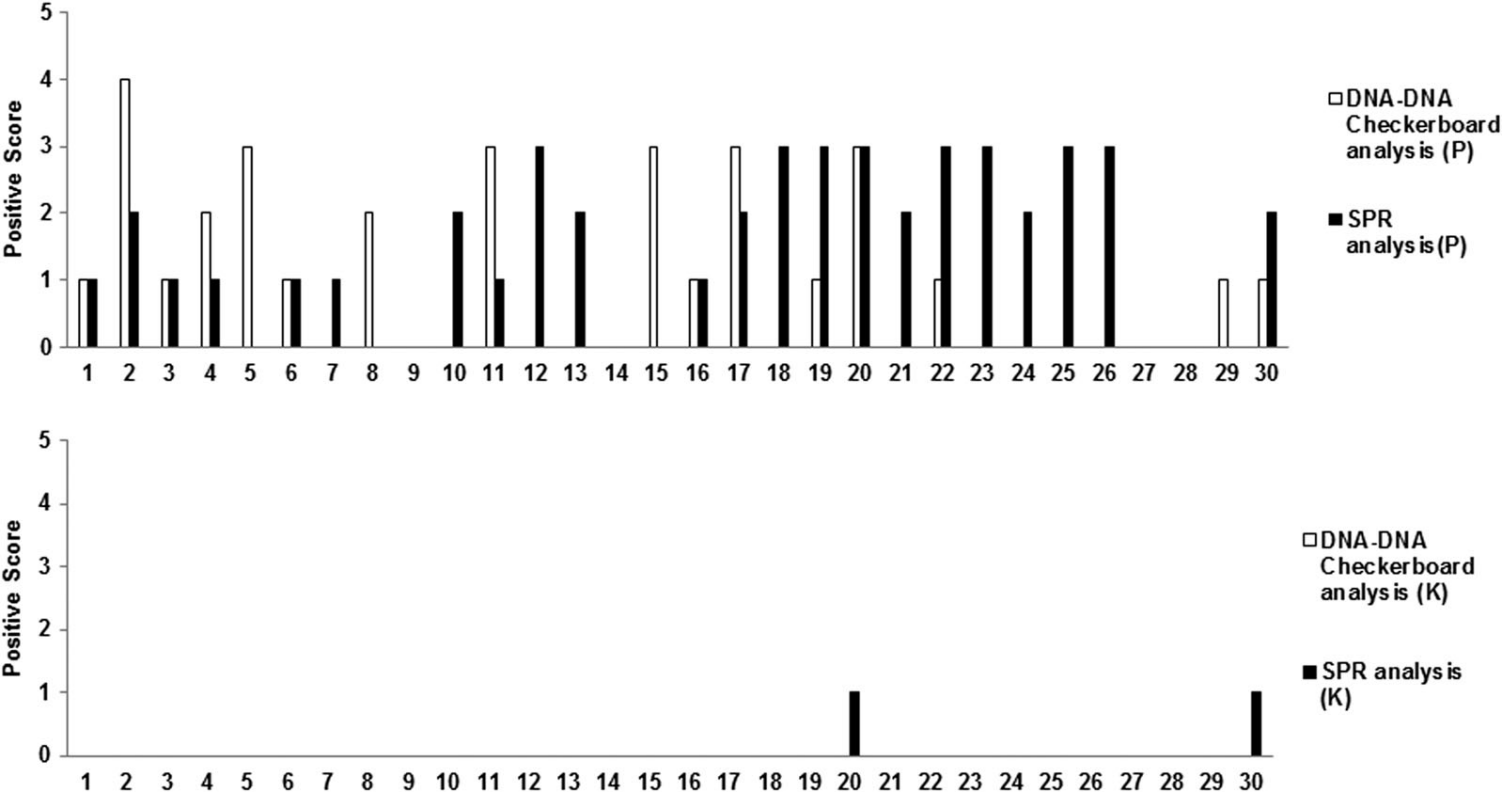
Detection of bacteria in clinical samples. Gingival crevicular fluid from healthy and chronic periodontitis patients was run in channels immobilized with in vitro anti‐*P*. *gingivalis* antibodies. Results obtained in surface plasmon resonance (SPR) analysis was compared to DNA–DNA checkerboard analysis (positive response unit [RU] in SPR = presence of ≥10,000 colonies in DNA–DNA checkerboard analysis). Chi square analysis of the results obtained by both methods (degree of freedom = 1) did not reveal a significant difference (*P* > 0.05, null hypothesis rejected implying no significant difference in results obtained in two methods used). *Y*‐axis is plotted with positivity (the positive response obtained in SPR analysis) as 100RU representing positivity of 1 and ≥10,000 colonies representing positivity of 1 due to variation in minimum detection limits in the two methods.

Gingival crevicular fluid samples from patients with periodontitis were analyzed for the presence of *P*. *gingivalis* using the channel immobilized with anti‐*P*. *gingivalis* antibodies produced in vitro. The results obtained in SPR analysis were compared with the results obtained using DNA–DNA checkerboard analysis (Fig. 8). Samples were considered positive if ≥10,000 bacterial cells were identified in the DNA–DNA checkerboard analysis and with a positive RU obtained in SPR analysis. A relative score of 100 is given for a positive response of ≥10,000 bacterial cells and 100RU, 200 for ≥20,000 bacterial cells and 200 RU, and so on. The results obtained in SPR analysis for detection of *P*. *gingivalis* in GCF samples were not significantly different from that obtained in DNA–DNA checkerboard analysis.

**Figure 9:**
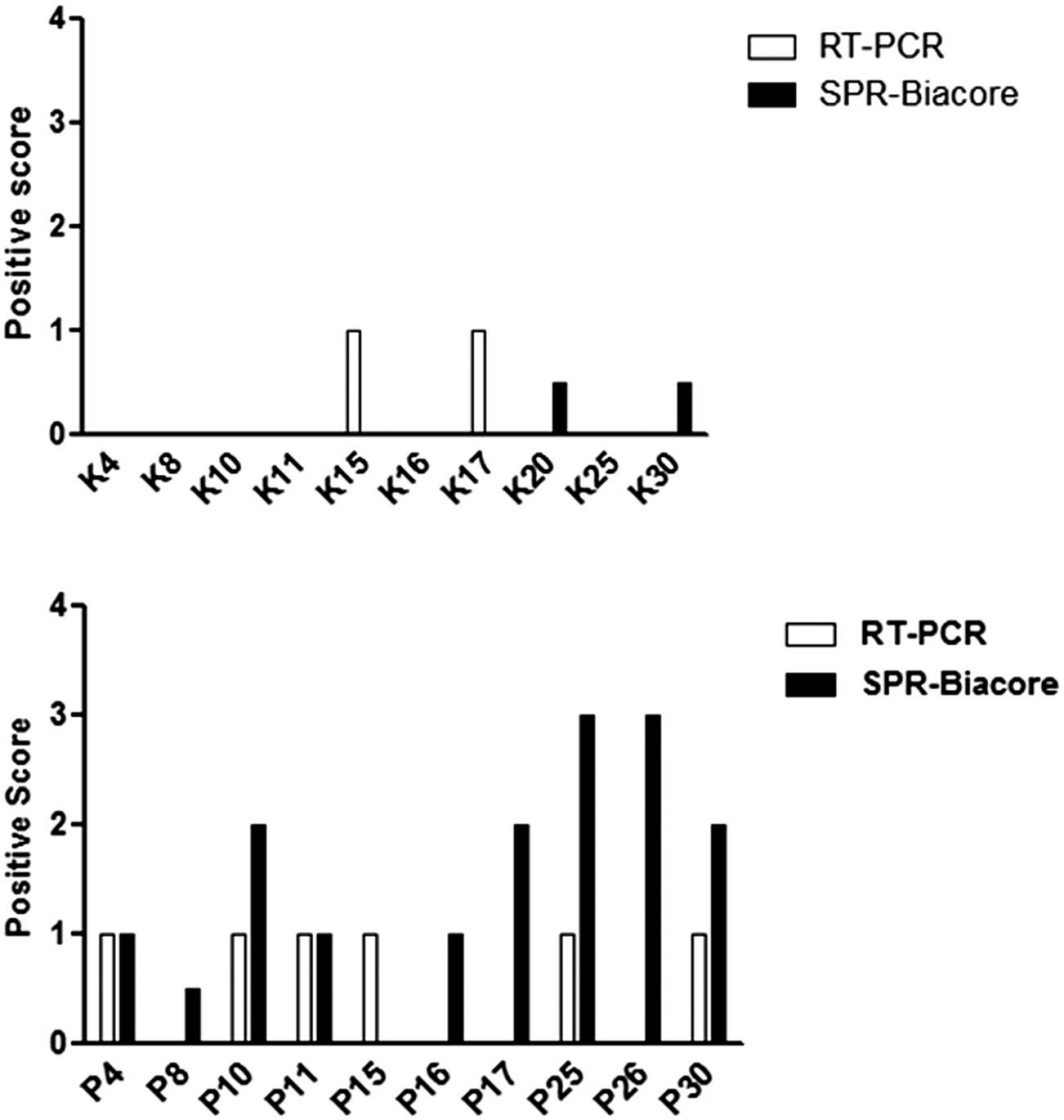
Scores for comparing reverse transcription‐PCR (RT‐PCR) and surface plasmon resonance (SPR) analysis (biacore) results for chi square analysis. A relative score for Ct values was given as 30–39 = 0; 20–29 = 1; and unamplified 0 = 0, while response obtained in SPR analysis was given a score as 300–399 RU = 3; 200–299 RU = 2; 100–199 RU = 1; and 1–99 RU = 0.5 to compare the results from both the methods using chi square analysis. From chi square distribution table, *P* > 0.05 was obtained for a degree of freedom 1 and 95%confidence interval; thereby, results obtained in both methods are not statistically different.

The RT‐PCR results also correlate with SPR analysis results (Fig. 9). Ct values obtained in RT‐PCR for GCF samples of healthy and patients were shown in Table 3, and a relative score was given to each of the Ct values obtained to compare to respective SPR analysis responses. Chi square analysis was performed, and no statistical differences were observed.

**Table 3:** RT‐PCR for detection of *P*. *gingivalis* in GCF samples

Controls (K)	Ct values	Patients (P)	Ct values
K4	30.67	P4	29.41
K8	30.28	P8	31.2
K10	33.32	P10	27.92
K11	34.96	P11	26.1
K15	26.03	P15	29.85
K16	0	P16	32.18
K17	29.51	P17	31.14
K20	30.2	P25	28.71
K25	30.52	P26	37.13
K30	31.68	P30	28.16

RT‐PCR, reverse transcription‐PCR; *P*. *gingivalis*, *Porphyromonas gingivalis*.

RT‐PCR was performed to detect *P*. *gingivalis* using gingival crevicular fluid (GCF) samples collected from healthy volunteers (*n* = 10) and also from patients with chronic periodontitis (*n* = 10). A total of 10 from 30 of controls and patient samples presented before in Figure 8 were chosen based on differences in responses (positive/negative and high/low values) obtained in biacore and DNA–DNA checkerboard analysis. Ct values obtained for *P*. *gingivalis* detection in GCF samples are presented.

### Attenuation of surface plasmon resonance response of bacterial strains

**Figure 10:**
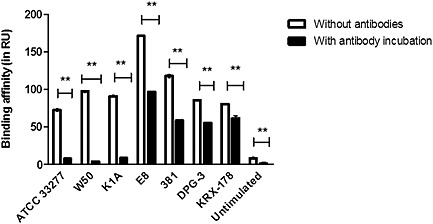
Incubation with anti‐*Porphyromonas gingivalis* antibodies attenuates the detection response. Detection of *P*. *gingivalis* strains in plasma samples collected after stimulating the whole blood of healthy donors with wild and mutant strains of *P*. *gingivalis* did not show a strain specific interaction, but the binding affinity was attenuated significantly on incubating the samples with anti‐*P*. *gingivalis* antibodies. ** The asterisks symbol refers to statistical significance with *P* < 0.01 by two‐way analysis of variance for (*n* = 3). RU, response unit.

Plasma samples were collected after stimulating the blood from healthy volunteers with *P*. *gingivalis* wild‐type and mutant strains. SPR analysis was performed with plasma samples incubated with anti‐*P*. *gingivalis* antibodies and was compared to samples without antibody incubation. Results showed that both wild type and mutant strains of *P*. *gingivalis* were able to bind to anti‐*P*. *gingivalis* antibodies but the response decreased upon incubation with antibodies (Fig. 10).

## Discussion

Antibodies against *P*. *gingivalis* developed in vitro during the study were used to detect bacteria in real time in clinical samples using SPR analysis. Naïve B‐cells offer few cell surface antibody receptors for specific antigen but exhibit a tremendous increase upon activation by any kind of stimuli (antigen) and develop into antibody‐secreting plasma cells (Williams and Williams, 2000). This study reports the in vitro survival of plasma cells and specificity of antibodies secreted in the presence of activated immune cells regulated in the presence of an infectious stimulant.

Lymphocytes were isolated from a donor who has been cured of periodontitis, and the choice of the donor was based on previous case history of >30,000 copies of *P*. *gingivalis* in GCF to ensure that the infection has passed through the system and would be able to process antigen presentation to secrete antibodies through memory repertoire. Previous studies (Ma and Hendershot, 2003; Tosato et al*.,* 1980; Whiteside and Rowlands, 1977) have shown changes in cell appearance during the maturation and differentiation of inactive B‐cells into plasma cells suggesting that the cell morphological changes observed was due to developmental stages of B lymphocytes in response to *P*. *gingivalis* stimulation in vitro.

Differentiation of B‐cells into antibody secreting plasma cells is mediated by T‐cells and other immune cells in vivo (Defrance et al*.,* 1992; Kosco et al*.,* 1989; Slifka and Ahmed, 1998), and therefore all the isolated peripheral blood mononuclear cells were used in the culture as such to aid different stages of B‐cell maturation. An increase in number of immune cells in the population after stimulation clearly revealed an activated immune response. Increased levels of IL‐6 and IL‐10 were observed in stimulated cell supernatants (data not shown), and it correlates with previous reports that IL‐6 and IL‐10 promote B‐cell differentiation (Itoh and Hirohata, 1995; Jego et al*.,* 2003). Stimulation of memory cells in an isolated and cultured lymphocyte population with antigens similar to the previous infections simplifies the process of antibody secretion. Furthermore, stimulated cell population showed the presence of plasma cells after 3 weeks, which could be because of increased dosage of bacterial stimulation that also provided a suitable environment with increased number of T‐cells that could mediate plasma cells survival in vitro (Fairfax et al*.,* 2008).

Subsequent invasion of pathogens or repeated stimulation with the same kind of antigen causes class switching in secreted immunoglobulins (Goldsby and Osborne, 2002). IgG is secreted at this phase, whereas IgM is secreted during the first exposure to an antigen (Byrne et al*.,* 2009). Previous molecular studies of immunoglobulin subclasses have shown that unreduced IgG molecules result in bands at 150 KDa in their impure state whereas the reduced form of IgG in its pure state results in bands at 50 and 25 KDa for heavy and light chains, respectively (Byrne et al*.,* 2009; Kosco et al*.,* 1989).

(Verma et al*.,* 2010) have shown that IgG subclasses could be used to understand the elicited host response (Th1 or Th2) against *P*. *gingivalis*. Further, increased IgG antibodies in patients with periodontitis contributed to increased tooth loss that correlated to elevated risk for coronary heart disease (Pussinen et al*.,* 2003). Anti‐*P*. *gingivalis* antibodies were of IgG class, and increased levels of IgG1 and IgG2 in our study correlate with previous studies, where increased IgG 1 and IgG 2 titers were observed on immunizing animals with *P*. *gingivalis* whole cells, cysteine proteases of *P*. *gingivalis* (Rajapakse et al*.,* 2002), and recombinant hemagglutinin B (Katz et al*.,* 1999).

Antigen‐antibody interactions have been studied for decades, and several immunoassay techniques, such as ELISA and immunosensor methods, are well established in recent years (Byrne et al*.,* 2009). Previous studies have used the SPR method to identify foodborne pathogens (Kobayashi et al*.,* 2009; Rich and Myszka, 2008). Biosensor immobilized with antibodies produced in vitro as described in this study could enable real‐time diagnosis in patients. Based on the results of the specificity test, we found that the antibodies were specific that could be due to high doses used for stimulation that could cause antigen‐specific B‐cell development as suggested by (Fairfax et al*.,* 2008). In addition, the antibodies developed were shown to be reliable for identifying specific bacteria such as *P*. *gingivalis* from clinical samples of patients with periodontitis. Similar results were obtained when detecting pathogens thriving within biofilms in leg ulcer patients using antibodies produced in vitro against common leg ulcer pathogens that live in biofilms (data not shown).

*Porphyromonas gingivalis* wild‐types (ATCC 33277, W 50, and 381) and mutant strains (E8‐ arginine gingipain mutant of W 50 and K1A‐lysine gingipain mutant of W 50, and major fimbriae mutant of 381‐DPG‐3 and minor fimbriae mutant of 381‐ KRX‐178) were used to elucidate the strain‐specific responses of anti‐*P*. *gingivalis* antibodies. There were no differences in responses in detection of the different strains from plasma samples suggesting that anti‐*P*. *gingivalis* antibodies developed from wild‐type ATCC 33277 strain were directed against common epitope of *P*. *gingivalis*. Further, the binding responses of different strains decreased when plasma samples were incubated with anti‐*P*. *gingivalis* antibodies suggesting attenuating effects of antibodies due to specific antigen‐antibody interaction and also pave way to elucidate their role in passive immunization against *P*. *gingivalis*‐mediated diseases.

Antibodies developed in vitro could be used for an immunobiosensor‐based real‐time detection of bacterial pathogens causing infectious diseases to prevent delayed treatment due to diagnosis based on time‐consuming bacterial culturing or other laborious, complex, and expensive methods. *P*. *gingivalis* antibodies developed in this study could thus be further used to detect *P*. *gingivalis* infection in patients and to identify the role of the bacterium in several periodontitis‐associated diseases.
